# ATRA enhances the bystander effect of suicide gene therapy driven by the specific promoter LEP 503 in human lens epithelial cells

**Published:** 2012-07-25

**Authors:** Jin Yang, Tian-Jin Liu, Yong-Xiang Jiang, Yi Lu

**Affiliations:** 1 Department of Ophthalmology, Eye and ENT Hospital, Fudan University, Shanghai, China; 2 Genetic Engineering Group, Institute of Biochemistry and Cell Biology, Chinese Academy of Science, Shanghai, China

## Abstract

**Purpose:**

To establish a novel, targeted lentivirus-mediated *LEP503*-HSV-tk/GCV suicide gene therapy system combined with all trans-retinoic acid (ATRA) for the inhibition of human lens epithelial cell (HLEC) proliferation and treatment of posterior capsular opacification (PCO) after cataract surgery; to estimate the enhancement of the bystander effect by ATRA; and to explore the role of Connexin43 (Cx43) mediated gap junctional intercellular communication (GJIC) in the bystander effect of the HSV-K/GCV system.

**Methods:**

A Lenti-*LEP503*-HSV-tk-EGFP vector was generated by cloning the lens-specific promoter *LEP503* (lens specific promoter 503) from genomic DNA of HLECs by PCR. The vector was then inserted into the promoter-less vector from lentivirus-based (*CMV*)-HSV-tk-EGFP. The expressional specificity of the *LEP503* promoter was assessed by investigating the expression of *EGFP* (enhanced green fluorescent protein) and *HSV*-*tk* (herpes simplex virus thymidine kinase) mRNA, both driven by Lenti-*LEP503*-HSV-tk-EGFP vector, by fluorescence microscopy, RT–PCR, flow cytometry, and western blot assays in HLECs, human adult retinal pigment epithelium cells (RPECs), human adult skin fibroblast cells (ASFCs), and Hela cells. Morphological changes were observed by fluorescence microscopy and cell viability was determined using the Cell Counting kit-8 Cell Proliferation (CCK-8) and MTT (3-(4,5-Dimethylthiazol-2-yl)-2,5-diphenyltetrazolium bromide) assays after Lenti-*LEP503*-HSV-tk/GCV system combined with ATRA treatment on HLECs. Flow cytometry, DNA fragmentation, and western blot assays were employed to analyze the mechanisms of bystander effects.

**Results:**

The promoter *LEP503*-mediated *HSV-tk* was specifically expressed in HLECs, and ATRA dose-dependently strengthened the bystander effect following *LEP503*-mediated HSV-tk/GCV gene therapy against lens cells by upregulating the expression of the gap junction protein Cx43.

**Conclusions:**

The Lenti-*LEP503*-HSV-tk/GCV suicide gene therapy system, combined with ATRA as an adjuvant, may be a feasible supplementary method for PCO treatment that targets residual lens cells.

## Introduction

Prodrug/suicide gene therapy that delivers a ‘suicide’ gene to target cells, and rendering them sensitive to a specific prodrug, is a promising strategy for treatment of malignant tumors [1]. Among the suicide gene therapy strategies, the Herpes Simplex Virus thymidine kinase (HSV-tk)/ganciclovir (GCV) is one of the best-characterized systems and has been successfully used in vitro and in vivo for treatment malignancies [2-5]. Theoretically, the *HSV-tk* gene, but not the mammalian form, can sensitize cells to the normally non-toxic antiviral drug, GCV. This occurs because GCV is selectively converted to its monophosphate by HSV-tk and then further phosphorylated by cellular kinases to its cytotoxic triphosphate derivative. This is then incorporated into cellular DNA and results in termination of DNA synthesis and cell death [2,6].

This suicide gene system has been explored as a treatment for other proliferative diseases [7,8] besides malignancies. In the eye, one promising potential target for this type of therapy is posterior capsular opacification (PCO). This disorder is the most common complication after cataract surgery and is caused by excessive proliferation of residual lens epithelial cells over the lens equator and onto the posterior lens capsule [9-11]. Currently, no effective means are available for complete eradication or removal of the residual lens epithelial cells during surgery [12,13]. Suicide gene therapy for lens epithelial cells is now being explored as a potential treatment for PCO and work from our own and other laboratories indicate that this strategy holds promise [9-11].

However, this drug sensitive gene treatment system has several limitations. One of the major limitations of current gene therapy is the non-selectivity of the *CMV* (cytomegalovirus) promoter, which causes the death of the lens epithelial cells, but also death of the corneal endothelial and iris pigmental epithelial cells [9-11]. A potentially more beneficial promoter for a suicide gene system is the lens specific promoter *LEP503* (lens epithelium gene product 503) [11,14]. This is a highly conserved gene involved in lens epithelial cell differentiation in different vertebrate species, and is localized in the epithelial cells along the entire anterior surface of the lens [15,16].

One of the most attractive elements in the HSVtk/GCV suicide system is the so-called “bystander effect” whereby cells that are not transduced with the *HSVtk* gene are also eliminated along with *HSVtk* gene-transduced cells. It is now well known that the efficiency of HSV-tk/GCV therapy is improved by this bystander effect, which relies on the ability of infected cells to kill neighboring uninfected targeting cells mainly through gap junction-mediated intercellular communication (GJIC) [17]. One ideal wide-spectrum chemical inducer of GJIC is trans-retinoic acid (ATRA), which results in upregulation of the expression of connexin43 (Cx43) and GJIC [17-19]. ATRA also plays an important role in regulation of cell growth and differentiation, especially in epithelial cells [20]. Some research has also indicated that ATRA, combined with other drugs, could enhance the therapeutic effects of the drugs [19-23]. However, no reports have yet described the effects of a HSV-tk/GCV system, driven by specific promoter *LEP503* and combined with ATRA, on human lens epithelial cell (HLEC) proliferation.

In our previous study, the toxic effects of the constitutive *CRV* promoter on the surrounding normal cells was avoided by insertion of the lens-specific promoter *LEP503* with an enhanced Cre recombinase (*Cre/loxP*) system to construct a *LEP503*-HSV-tk/GCV vector. The vector specifically drove HSV-tk expression in HLECs and the LEP503 promoter restricted expression of the reported gene to the lens cells [11] in agreement with in vivo findings [16]. However,the promoter inserted into the HSV-tk/GCV vector did not provide high levels of expression. We have been endeavoring to improve the therapeutic efficacy of this system through different clone strategies, use of special promoters, and in combination with other reagents. In the present study, we describe a simple and feasible strategy that involves the construction of a lentivirus-mediated *LEP503*-HSV-tk/GCV system. Importantly, ATRA dramatically increased the sensitivity of HLECs to GCV in this *LEP503*-HSV-tk/GCV system, and also significantly strengthened the bystander effect, possibly through upregulation of Cx43 expression. These observations suggest that suicide gene therapy driven by the lens epithelial cells-specific promoter of *LEP503,* combined with ATRA as an adjuvant, may be a feasible strategy for PCO treatment.

## Methods

### Construction of recombinant lentiviral vectors

We amplified the *LEP503* promoter with KOD-PLUS kit (Toyobo, Osaka, Japan) according to the manufacturer’s procedure guide. A volume of 1×10^6^ HLECs was collected and lysed, then genomic DNA was isolated with Viogene VioFast Blood & Cell Genomic DNA Extraction Minprep System (Viogene-Biotek Corporation, Taibei, China) according to the manufacturer's instructions. The *LEP503* promoter was cloned from HLEC genomic DNA by PCR, using the following set of primers: forward primer 5′-atc gat ctc cag cct ggg caa caa aac aag a-3′ and reversed primer 5′-gga tcc gtg ggc aac agt tcc gag gaa ggg t-3′. The products contained Cla I site at their 5′-ends and BamH I site at their 3′-ends. The amplification conditions were as follows: 94 °C for 2 min, 40 cycles of 94 °C for 15 s, 59 °C for 2 min and 68 °C for 2 min; and finally 72 °C for 10 min. The PCR products, 2,439 base pairs (bp), were incompletely double-digested with Cla I and BamH I restriction enzymes. The digested products (2,436 bp) were ligated into the promoter-less vector double-digested with Cla I and BamH I enzymes from Lenti-CMV-HSV-tk-EGFP, which has been constructed as previously described [10,11]. The recombinant lentiviral vector (Lenti-*LEP503*-HSV-tk-EGFP) was sequenced and the sequence was used to BLAST search in gene bank to confirm the nucleotide constitution.

### Lentiviral vector preparation and virus infection

As previously described [10,11], the transfer vectors and the packaging system pSPAX2 and pMD2.G vectors (kindly provided by Dr. Didier Trono, University of Geneva, Geneva, Switzerland) were produced by transient transfection into 293T cells. Around 5×10^6^ 293T cells were seeded onto 10-cm-diameter dishes 24 h before transfection. A total of 20 μg of plasmid DNA was used to transfect one dish: 5 μg of the envelope plasmid pMD2.G, 5 μg of packaging plasmid pSPAX2, and 10 μg of transfer vector plasmid (*LEP503*-HSV-tk-EGFP or *CMV*-HSV-tk-EGFP). Precipitates were formed by adding the plasmids to: 250 µl 0.5 M CaCl_2_ and 0.1× TE (pH, 8.0), final volume is 500 μl, mixing well, then adding 500 μl of 2× HEPES-buffered saline dropwise containing 281 mM NaCl, 50 mM HEPES, and 1,500 mM Na_2_HPO_4_ (pH 7.12), while vortexing and immediately adding the precipitate to the cultures. The medium (10 ml) was replaced 14~16 h after transfection. The conditioned medium was collected after another 24 h and cleared by low-speed centrifugation, followed by filtration through 0.45-μm-pore-size cellulose acetate filters. Virus was collected by ultracentrifugation for 90 min at 80,000× g at 4 °C. The pellet was re-suspended in 1 ml phosphate buffered saline (PBS) and stored at −80 °C. The concentrated supernatants were titered by serial dilutions of vector stocks on 1×10^5^ Hela cells, followed by fluorescence-activated cytometric analysis according to the formula: 1×10^5^ Hela cell × %EGFP positive cell × 1,000/μl virus. Titers of lentiviral vectors were 1×10^8^ ~1×10^9^ TU/ml.

### Cell culture

The HLEC line SRA 01/04 (transformed by large T antigen), human adult retinal pigment epithelium cell line D407 (RPECs), HeLa, and 293T cell strains were purchased from the American Type culture Collection (ATCC, Manassas, VA). Human adult skin fibroblast cells (ASFCs) were a gift from the central laboratory of the Shanghai Eye and ENT Hospital, and these cells were cultured in DMEM (Invitrogen, Grand Island, NY) with 10% FBS (Invitrogen). The expression of EGFP was analyzed by plating cells at 2×10^5^ cells/well in six-well plates. After cells adhesion, the lenti-*CMV*-HSV-tk-EGFP or lenti-*LEP503*-HSV-tk-EGFP vectors were added (the multiplicity of infection [MOI] was 10 and 40, respectively). polybrene (Sigma, St. Louis, MO; 8 µg/ml) was added to all lentiviral vectors infections dishes. Transfection efficiency was determined by visualizing EGFP (enhanced green fluorescent protein) expression by fluorescence microphotography or by western blot assays. The mRNA transcription of HSV-tk was analyzed by RT–PCR assay, after infected cells were cultured for 48 or 72 h. The cytotoxicity of GCV (InvivoGen, San Diego, CA) was evaluated by plating cells into six-well dishes at a density of 5×10^5^ cells/well and incubating at 37 °C in 5% CO_2_ for 24 h. The cells were then infected with lenti-*LEP503*-HSV-tk-EGFP and lenti-*CMV*-HSV-tk-EGFP (MOI=40 and 10, respectively). Twenty-four hours after infection, cells were treated with GCV (20 µM) and incubated at 37 °C in 5% CO_2_. Morphological changes were observed by fluorescence microscopy and cell viability was determined using the Cell Counting Kit-8 Cell Proliferation Assay (CCK-8; Dojindo, Kumamoto, Japan) or the MTT assay at 12, 24, 36, 48, 60, and 72 h after treatment.

The in vitro cell killing effect was investigated by seeding HLECs infected by lenti-LEP503-HSV-tk-EGFP at a density of 5×10^3^ cells/well in 96-well plates and incubating for 24 h. The cells were then exposed to GCV (ganciclovir; 20 µM) and incubated at 37 °C in 5% CO_2_ for 12, 24, 36, 48, 60, and 72 h. The bystander effect was assayed with the CCK-8 (cell counting kit-8 cell proliferation) or MTT ( 3-(4,5-Dimethylthiazol-2-yl)-2,5-diphenyltetrazolium bromide) assays. For co-culture, 24 h after being infected with lenti-*LEP503*-HSV-tk-EGFP vector, HLECs were trypsinized and mixed with uninfected cells at ratios of 0, 20, 40, 60, and 80%, and then were re-plated in 24-well culture plates. The cells were simultaneously exposed to 20 μM GCV, 10^−7^ M ATRA (all trans-retinotic acid), and (or) 40 μM 18-α-glycyrrhetinic acid (18-α-GA; Sigma-Aldrich, St. Louis, MO) for 72 h.

### RNA extraction and RT–PCR analysis

Three weeks after stable expression of HSV-tk was observed in HLECs and Hela cells, total RNA was extracted from HLECs and Hela cells and reverse transcribed according to the protocol described for the RT–PCR kit (Promega, Madison, WI). Relative transcription of HSV-tk and EGFP was determined by performing a semi-quantitative PCR for *HSV-tk* and β-actin (*ACTB*; internal control) analysis with PCR amplification for 28 cycles. For *HSV-tk*, the forward primer was 5′-atc cca tcg ccg ccc tcc tgt gc-3′ and the reverse primer was 5′-cgc ccc cga aag ctg tcc cca atc-3′, for *EGFP*, the forward primer was 5′-cga gct gga cgg cga cgt aaa c-3′ and the reverse primer was 5′-gcg ctt ctc gtt ggg gtc ttt g-3′, and for *ACTB*, the forward primer was 5′-aac gag cgg ttc cga tgc cct gag-3′ and the reverse primer was 5′-tgt cgc ctt cac cgt tcc agt t-3′. The amplification conditions were as follows: 94 °C for 5 min, 28 cycles of 94 °C for 1 min, 58 °C for 2 min and 72 °C for 1 min; and final 72 °C for 10 min. The expected lengths of the *HSV-tk*, *EGFP*, and *ACTB* products were 613, 597, and 590 bp, respectively. A total of 10 µl of each PCR product was loaded onto a 1.5% agarose gel containing 0.5 µg/ml of ethidium bromide and separated by electrophoresis. The expression differences between *HSV-tk* and *EGFP* were quantified with Image-Pro Plus 5.0 software (Media Cybernetics, Silver Spring, MD) and normalized with respect to *ACTB* expression.

### Protein collection and western blot analysis

Protein expression of Cx43 was analyzed by seeding 1×10^6^ HLECs in 25 mm^2^ cell culture flasks for 24 h and then exposing them to 10^−7^ mol ATRA l^−1^. The cells were collected and lysed with RIPA buffer including protease inhibitor cocktail (Pierce, Rockford, IL) at 0, 12, 24, 36, and 48 h after ATRA treatment, or at 72 h after exposure to different doses of 18-α-GA. EGFP expression was analyzed by collecting and lysing the cells with the same RIPA buffer at 0, 24, 48, and 72 h after infection. A total of 100 μg cell protein were separated by standard sodium dodecyl sulfate–PAGE under reducing conditions and blotted to polyvinylidene difluoride membranes using a semidry blotting apparatus. After blocking (0.5% defatted dry milk in TBS with 0.1% Tween-20 (TBST)), the membranes were incubated with the primary monoclonal mouse anti-Cx43 antibody (1: 2000; Sigma), goat anti-EGFP (1:1,000; Abcam, San Francisco, CA), mouse anti-GAPDH antibody (1:5,000; Santa Cruz Biotechnology, Santa Cruz, CA), and mouse anti-β-actin (1:5,000; Santa Cruz Biotechnology) in blocking buffer for 1 h, and were washed three times with TBST. The secondary peroxidase-conjugated donkey anti-mouse or goat antibodies in TBST were added for 1 h. Finally, the members were washed with TBST for 20 min, and immunoreactive proteins were visualized using the Western Blot Chemiluminescence System (Amersham™ ECL; GE Healthcare, Little Chalfont, Buckinghamshire, UK). Primary and secondary antibodies were diluted in 5% defatted dry milk in TBST. The spot densities for EGFP, Cx43, β-actin, and GAPDH were quantified with Image-Pro Plus 5.0 software and normalized by the respective GAPDH or β-actin.

### Flow cytometry analysis

The specificity of the *LEP503* promoter was analyzed by trypsinizing and collecting HLECs, RPECs and ASFCs 48 h after infection with lenti-*LEP503*-HSV-tk-EGFP (MOI=50) or lenti-*CMV*-HSV-tk-EGFP vector (MOI=5). Cells were washed with cold PBS and analyzed by flow cytometry (Beckman LSRII; Beckman Coulter, EI Cajon, CA). For analysis of cell death, cells were infected with lenti-*LEP503*-HSV-tk-EGFP or lenti-*CMV*-HSV-tk-EGFP vector for 24 h and following exposure to GCV (20 μM) and ATRA (10^−7^ M) for 72 h. Cells were then trypsinized, collected, and re-suspended in cold PBS (10^6^ cells/ml). After washing twice with cold PBS, cells were re-suspended in 100 μl binding buffer, then 2 μl Annexin-V-APC (20 μg/ml) was added and the cells were incubated for 15 min on ice. Then samples were transferred into detective tubes and 400 μl PBS was added. Cellular DNA was stained with 1 μl propidium iodide (PI, 50 μg/ml) for 2 min. Cell necrosis and apoptosis were determined by flow cytometry (Beckman LSRII). Cells without Annexin-V-APC and PI treatment were used as blank controls.

### CCK-8 and MTT assay

Time- and dose-dependent cell viability was monitored using CCK-8 or MTT assay. CCK-8 is a sensitive nonradioactive colorimetric assay for determining the number of viable cells in cell proliferation and cytotoxicity assays. The solution is added directly to the cells and no pre-mixing of components is required. CCK-8 uses Dojindo's tetrazolium salt, WST-8[2-(2-methoxy-4-nitrophenyl)-3-(4-nitrophenyl)-5-(2, 4-disul-fophenyl)-2H-tetrazolium, monosodium salt], which produces a water-soluble formazan upon reduction in the presence of an electron carrier. The amount of yellow formazan produced in cells is directly proportional to the number of living cells. Briefly, at the end of the culture period, 10 μl of the CCK-8 solution was added to each well of the culture plate. After a 4-h incubation, absorbance at 450 nm was measured with a universal microplate spectrophotometer (Biotek Instruments, Gene Company Limited, Winooski, VT). The cytotoxic effect was indicated as a percentage of surviving cells (ratio of surviving cells after treatment and without treatment) using the following formula: Cell viability=(absorption of sample - absorption of background)/ (absorption of control - absorption of background) × 100%.

For the MTT assay, 20 μl MTT stock solution (0.5 mg/ml) was added to cells in each well of a 96-well tissue culture plate, which was then incubated at 37 °C for 4 h. The medium containing MTT was removed and then 200 μl DMSO (DMSO) was added per well to stop the reaction and dissolve the formazan crystals. After 15 min, the absorbance in each well was measured at 570 nm using the universal microplate spectrophotometer (Gene Company). Cultures without cells were used as blank controls. The percentage of surviving cells was calculated using the following formula: Cell viability=(absorption of sample - absorption of background)/ (absorption of control - absorption of background) × 100%.

### Immunocytochemistry

Cells (seeded on cover glasses) were fixed with 4% paraformaldehyde for 15 min at room temperature then washed 3 times (3 min/per time) with PBS. Cells were perforated with 0.5% Triton X*-*100 in PBS for 15 min and then blocked in PBS containing 10% goat serum, 0.1% gelatin and 0.05% Triton X*-*100 for 30 min. Cells were incubated overnight at 4 °C with mouse antibodies against Cx43 (1:1,000; Sigma). After washing 3 times with PBS, cells were incubated for 30 min at 37 °C with Cy3-conjugated goat anti-mouse secondary antibodies. The nuclei were counterstained with DAPI (4,6-diamino-2-phenyl indole).

### DNA rragmentation assay

Cells (1×10^6^) infected with Lenti-LEP503-HSV-tk-EGFP or Lenti-*CMV*-HSV-tk-EGFP were exposed to 20 µg GCV ml^−1^. A sufficient number of cells (1×10^7^) were collected at 72 h and washed in PBS before resuspension in 1 ml of fresh lysis buffer (50 mM, pH 8 Tris, 100 mM EDTA, 1% SDS, 100 μg proteinase K ml^−1^, and 1 mM NaCl). Samples were incubated at 37 °C for 16 h with gentle rocking. The DNA was extracted with an equal volume of phenol/chloroform/isoamyl alcohol solution (25:24:1) and precipitated with 3 M sodium acetate (pH 5.2) and 100% ice-cold ethanol. The pellets were dried by evaporation for 10 min, and resuspended in T-E buffer (10 mM Tris-HCl and 1 mM EDTA, pH 8.0) overnight at room temperature. The DNA was quantified by ultraviolet absorbance at 260 nm. Approximately 10 μg of DNA was added to each well after mixing with 5 μl loading buffer. Horizontal electrophoresis of DNA was performed for 2 h at 75 V on 1.5% agarose gels.

### Statistical analysis

All data were expressed as means±SD and analyzed with SPSS version 16.0. A Dunnett *t*-test and a one-way ANOVA were performed to assess the statistical significance between different groups. Significance for all tests was established at p<0.05.

## Results

### *LEP503* promoter mediated bicistronic lentiviral vector construction

**Figure 1:**
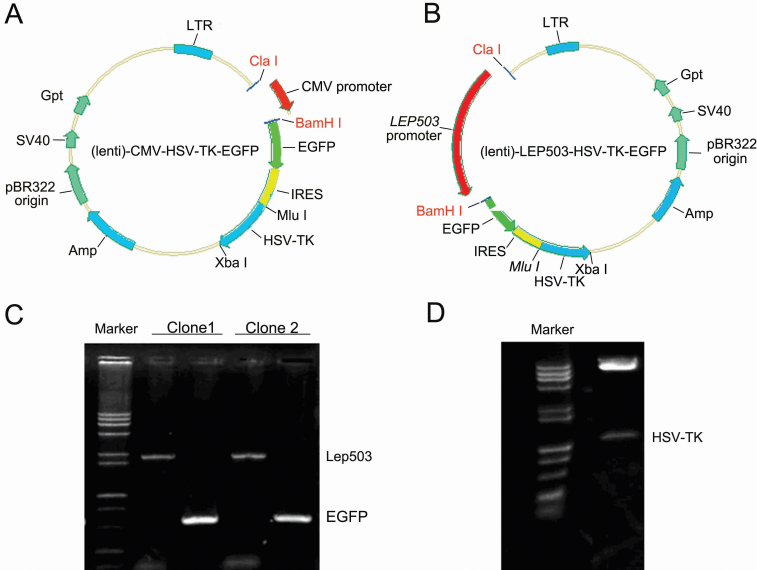
Construction of the (Lenti)-*LEP503*-HSV-tk-EGFP vector. **A**: The assumption diagram for the (Lenti)-*CMV*-HSV-tk-EGFP vector. **B**: The assumption diagram for the (Lenti)-*LEP503*-HSV-tk-EGFP vector. **C**, **D**: Confirmation of the (Lenti)-*LEP503*-HSV-tk-EGFP vector with a PCR assay (**C**) and an enzyme cut assay (**D**).

We generated the target plasmid using conventional molecular biologic techniques. First, we cloned the *LEP503* promoter from the mRNA of HLECs by RT–PCR, and designed the full length of *LEP503* (2,439 bp) containing double enzyme sites of Cla I and BamH I (Figure 1B). The digested product of *LEP503* (Figure 1B) was ligated onto the promoter-less vector lenti-*(CMV)*-HSV-tk-EGFP, which was also digested by Cla I and BamH I (Figure 1A). The recombinant lentiviral-*LEP503*-HSV-tk-EGFP vector was sequenced and the assumption diagram of the vector was showed in Figure 1B.

We verified whether the *LEP503* promoter was subcloned into the lenti-*LEP503*-HSV-tk-EGFP vector by first amplifying the *LEP503* promoter and EGFP with the PCR assay using the lenti-*LEP503*-HSV-tk-EGFP vector as the template. The full length of *LEP503* product, using its previous clonal primers (2,439 bp) and the EGFP product (597 bp) were detected by agarose gel electrophoresis (Figure 1C**)**. Second, the lenti-*LEP503*-HSV-tk-EGFP vector was further confirmed by double-digestion with Mlu I and Xba I. We found that the fragment of HSV-TK (~900 bp) was digested from the lenti-*LEP503*-HSV-tk-EGFP vector (Figure 1D). DNA sequencing, PCR results, and analysis of restriction enzyme digests verified the correct orientation of the insert in the constructs; i.e., the human *LEP503* promoter was subcloned into the expressional vector of lenti-HSV-tk-EGFP.

### *LEP503* promoter specifically drives the downstream targeting genes in HLECs

**Figure 2:**
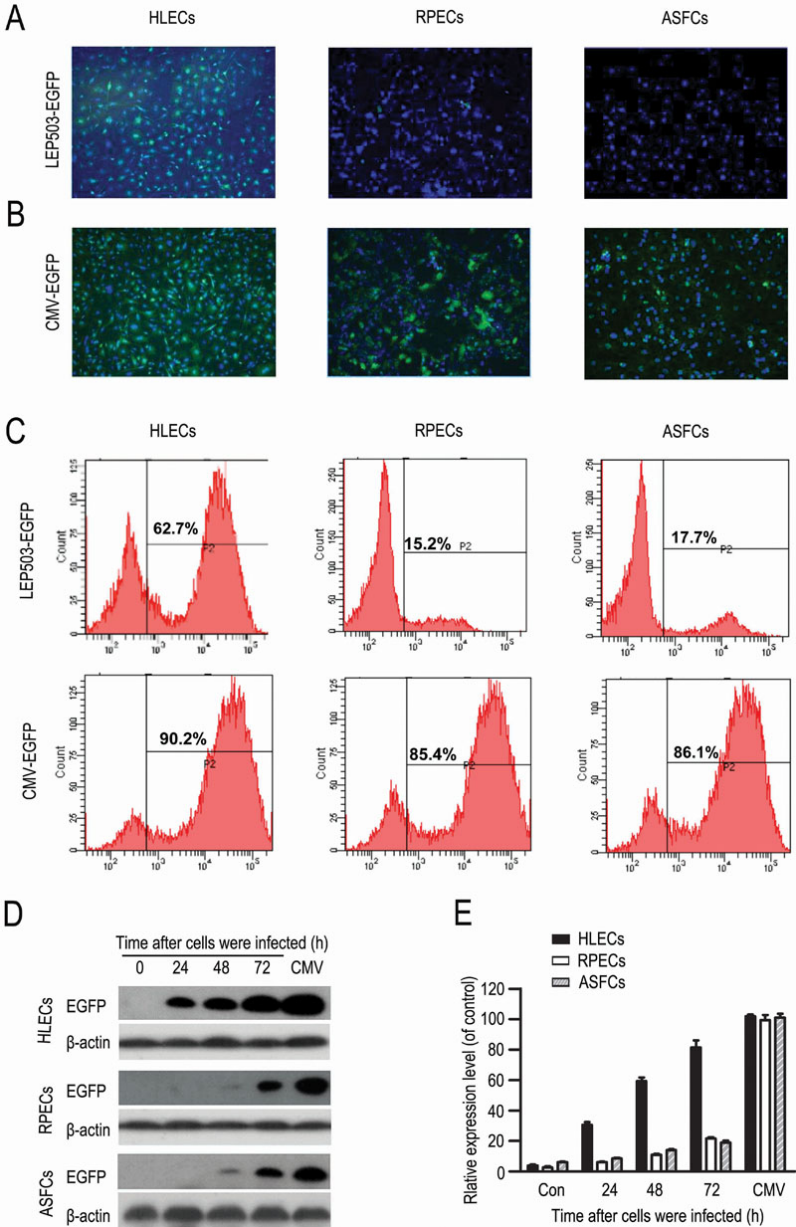
Analysis of LEP503 promoter specificity. **A**: The expression of EGFP driven by lenti-*LEP503*-HSV-tk-EGFP vector in HLECs, RPECs, and ASFCs at 72 h after being infected. **B**: The expression of EGFP driven by lenti-*CMV*-HSV-tk-EGFP vector in HLECs, RPECs, and ASFCs at 72 h after being infected. Magnification: 40×; Green: EGFP, Blue: DAPI staining. **C**: Flow cytometry evaluation of the expression of EGFP driven by lenti-*LEP503*-HSV-tk-EGFP and lenti-*CMV*-HSV-tk-EGFP vectors in HLECs, RPECs, and ASFCs at 48 h after being infected. **D**: western blotting assessment of the expression of EGFP driven by lenti-*LEP503*-HSV-tk-EGFP vector at 24, 48, and 72 h after being infected, and by lenti-*CMV*-HSV-tk-EGFP vector 72 h after being infected in HLECs, RPECs, and ASFCs. **E**: Semi-quantitative analysis of the western blotting results. The intensities of *HSV-tk* mRNA were normalized to *ACTB* (n=3).

**Figure 3:**
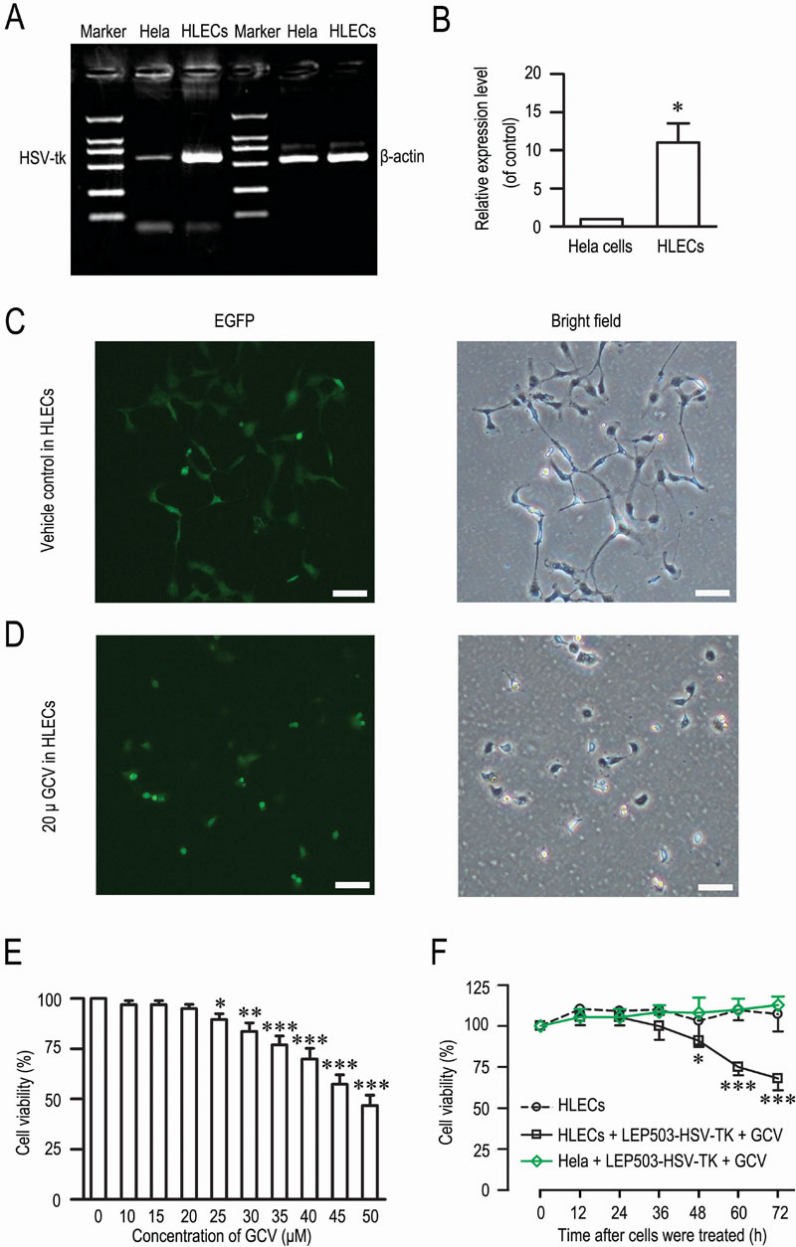
The inhibitory effects of the lenti-*LEP503*-HSV-tk/GCV system on HLECs. **A**: The transcriptional detection of *HSV-tk* mRNA driven by lenti-*LEP503*-HSV-tk-EGFP vector in Hela cells (left) and HLECs (right). *ACTB* (590 bp) was used as the internal control. **B**: Semi-quantitative analysis for the RT–PCR results (n=3). The intensities of *HSV-tk* mRNA were normalized to *ACTB* (n=3). **C**, **D**: Cell survival analysis by EGFP expression in HLECs 24 h after infection, followed 48 h by vehicle control treatment (**C**) and by GCV treatment (**D**). Scale bar: 100 μm. **E**: Cell survival analysis after exposure to a series of dose of GCV with MTT assay (n=5). **F**: The time-effect curve of the lenti-*LEP503*-HSV-tk/GCV system on HLECs and Hela cells with the CCK-8 assay (n=4). * p<0.05 and *** p<0.001, compared with Hela cell group (**B**), vehicle control (0; **E**) and control time point (**F**).

We assessed the relative specificity of the *LEP503* promoter by infection of the lenti-*LEP503*-HSV-tk-EGFP and lenti-*CMV*-HSV-tk-EGFP vectors into human RPECs, ASFCs, Hela cells, and HLECs. We found that significantly more EGFP-positive cells were observed in HLECs than in RPECs and ASFCs as determined by fluorescence microscopy at 72 h after infection by lenti-*LEP503*-HSV-tk-EGFP (Figure 2A), whereas more EGFP-positive cells were observed in HLECs, RPECs, and ASFCs after infection by lenti-*CMV*-HSV-tk-EGFP (Figure 2B). We further confirmed the relative specificity of the *LEP503* promoter in HLECs by detection of expression of EGFP protein with flow cytometry and western blotting assays, and the mRNA transcription of *HSV-tk* with RT–PCR assay (product: ~613 bp). As shown in Figure 2C, the percentages of *LEP503*-driven EGFP*-*positive cells in total cells were 62.7% in HLECs, 15.2% in RPECs, and 17.7% in ASFCs at 48 h after infection, whereas those of *CMV*-driven EGFP-positive cells were 90.2% in HLECS, 85.4% in RPECs, and 86.1% in ASFCs at 48 h after infection (Figure 2C). Western blotting results showed that the time-course of EGFP expression driven by *LEP503* promoter was substantially more rapid in HLECs than in RPECs and ASFCs (Figure 2D,E). In addition, we found that the mRNA transcription of *HSV-tk* driven by *LEP503* promoter was ~10 fold higher in HLECs than in Hela cells (p<0.05; Figure 3A,B). These findings indicated that the *LEP503* promoter could specifically initiate the transcription of downstream genes including *EGFP* and *HSV-tk* in HLECs. However, we found that, like most other tissue specific promoters used in gene therapy, the *LEP503* promoter mediated lower expression than some widely expressed promoters such as the *CMV* promoter (Figure 2), consistent with our previous report [11]. These results suggested that the *LEP503* promoter can specifically express the downstream operating genes in HLECs, but it has a lower promoter capability than the *CMV* promoter.

### Lenti-*LEP503-*HSV-tk-EGFP/GCV system inhibits HLECs proliferation

Next, we evaluated the effect of lenti-*LEP503*-HSV-tk-EGFP/GCV system on HLECsby infecting HLECs with the vector at a MOI=10 and lenti-*CMV*-HSV-tk-EGFP at MOI=5. After these cells were infected for 24 h and exposed to GCV (20 μM) for 48 h, we observed fewer EGFP-positive cells and most EGFP-positive cells were shrunken and floating (Figure 3D), whereas the vehicle control contained more EGFP-positive cells (Figure 3C). We assessed the cytotoxicity of GCV with the MTT assay after these cells were infected for 24 h and exposed to GCV (from 0~50 μM) for 72 h. We found an obvious cytotoxicity at doses of GCV 25 μM and higher (Figure 3E), but 20 μM GCV had no cytotoxicity for HLECs . We further measured the proliferation of cells after infection for 24 h, HLECs and Hela cells were exposed to GCV (20 μM) and viability was tested with the CCK-8 assay. The time course of the inhibitory effects of the system on HLECs and Hela cells is shown in Figure 3F. The kill effects were ~10, ~25, and ~32% at 36, 48, and 72 h after GCV treatment, respectively, whereas the system had no inhibitory effects on Hela cells (Figure 3F). These results indicated that the lenti-*LEP503*-HSV-tk/GCV system specially induced an inhibitory effect in HLECs and that the inhibitory effect was not directly produced by cytotoxicity of GCV itself.

### ATRA enhances the proliferative inhibition effect of lenti-*LEP503*-HSV-tk/GCV system against HLECs

**Figure 4:**
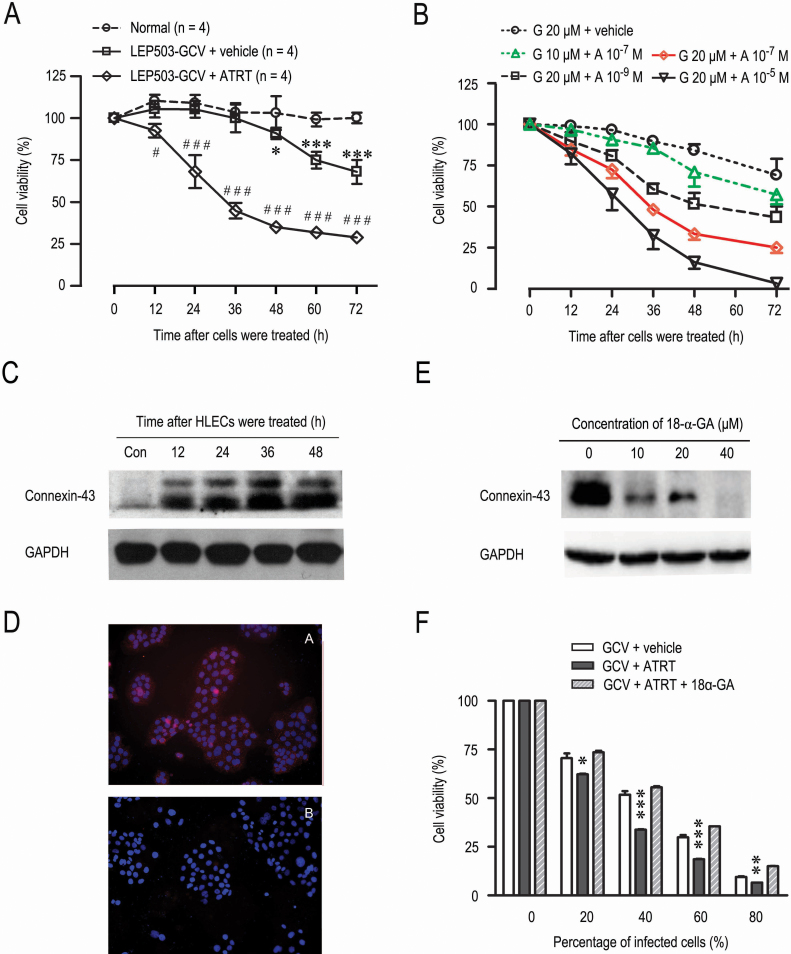
Cytotoxic effects of a combined treatment of lenti-*LEP503*-HSV-tk/GCV system and ATRA on HLECs. **A**: The time-effect curves of the lenti-*LEP503*-HSV-tk/GCV system combined with ATRA or vehicle treatment on HLECs with the CCK-8 assay (n=4). *LEP503*-GCV, lenti-*LEP503*-HSV-tk/GCV + vehicle treatment; *LEP503*-GCV + ATRT, lenti-*LEP503*-HSV-tk/GCV + ATRT treatment. **B**: Dose-dependent analysis of the cytotoxicity of lenti-*LEP503*-HSV-tk/GCV system combined with ATRA treatment on HLECs with the MTT assay (n=5). **C**: The expression of Cx43 in HLECs treated by lenti-*LEP503*-HSV-tk/GCV system combined with ATRA for 72 h with western blotting assay. GAPDH was used as the loading control. **D**: Immunostaining for Cx43 (red) in HLECs at 72 h after being treated by lenti-*LEP503*-HSV-tk/GCV system combined with ATRA (top) or with vehicle (bottom). Blue, DAPI staining. **E**: The expression of Cx43 in HLECs at 72 h after being exposed to 18-α-GA by western blotting assay. GAPDH was used as the loading control. **F**: Co-culture experiments to evaluate bystander killing and the inhibitory effects of 18-α-GA (40 μM) on the bystander effect in different proportions cells infected by lenti-*LEP503*-HSV-tk/GCV system with CCK-8 assay (n=3). * p<0.05, ** p<0.01 and *** p<0.001, compared with normal control; ^#^ p<0.05 and ^# # #^ p<0.001, compared with *LEP503*-GCV group.

We next determined whether ARTA affected the observed inhibition of HLEC proliferation. We assessed the bystander effect of the system by infecting HLECs with Lenti-*LEP503*-HSV-tk-EGFP and exposing them to GCV or (and) ATRA. The CCK-8 assays showed ~8, ~32, ~55, ~65, ~68, and ~71% inhibition at 12, 24, 36, 48, and 72 h after GCV and ATRA treatment, respectively, and ~0, ~0, ~10, ~25, and ~32% at 12, 24, 36, 48, and 72 h after GCV and vehicle control treatment, respectively (Figure 4A). We further confirmed the inhibitory effects using the MTT assay, the classical approach for measuring cell proliferation. We found that ATRA could dose-dependently enhance the killing effect of lenti-*LEP503*-HSV-tk/GCV system (Figure 4B) and that the inhibitory effect at 20 μM GCV and ATRT was similar to that observed with the CCK-8 assay (Figure 4A). These data indicate that the treatment with ATRA exerted a strong bystander effect when compared to the vehicle control treatment (Figure 4A,B).

The GJIC is well known as the main mechanism mediating the bystander effect in HSV-tk gene therapy. ATRA has been shown to upregulate the expression of Cx43 and GJIC [21-23]. Therefore, we evaluated the expression of the Cx43 by western blotting assay with a specific monoclonal antibody. As shown in Figure 4C, the combined lenti-*LEP503*-HSV-tk-EGFP/GCV system and ATRA treatment increased the time-dependent expression of Cx43 in HLECs. Semi-quantitative analysis showed that the Cx43 expressions were ~7, ~8, ~15, and ~16 fold higher than control values at 12, 24, 36, and 48 h, respectively, after the combined treatment with lenti-*LEP503*-HSV-tk-EGFP/GCV and ATRA (data not shown). We used immunostaining assay to confirm Cx43 expression in HLECs and found significantly higher expression at 72 h after the combined treatment with the lenti-*LEP503*-HSV-tk-EGFP/GCV system and ATRA when compared to the control (Figure 4D). These findings indicated that ATRA enhanced the expression level of Cx43.

The bystander effect was further evaluated by using the CJIC inhibitor 18-α-GA, which can effectively down regulate the expression of Cx43. We mixed HELCs infected with the lenti-*LEP503*-HSV-tk-EGFP vector with normal cultured HELCs at ratios of 0, 20, 40, 60, and 80%, and then exposed them to GCV, ATRT and 18-α-GA. As shown in Figure 4F, the killing effect of the combined treatment of lenti-*LEP503*-HSV-tk-EGFP/GCV system and ATRA was ~38, ~66, ~81, and ~94% for a ratio of 20, 40, 60, and 80% infected cells, respectively, whereas the killing effect of treatment with the lenti-*LEP503*-HSV-tk-EGFP/GCV system alone was ~29, ~48, ~70, and ~89%, respectively (Figure 4F). In addition, we found that a higher ratio of infected cells in the co-culture produced a greater bystander effect, and the killing efficiency percentage (~38, ~66, ~81, and ~94%) was significantly higher than the for increasing ratios of mixture infected cells to coculture cells (20, 40, 60, and 80%, respectively; Figure 4F), in support of a bystander effect. Notably, when these cells were incubated with the agent, 18-α-GA, a selective inhibitor of gap junctions, the expression of Cx43 was dose-dependently inhibited by 18-α-GA (Figure 4E). We also found that the bystander effect mediated by Cx43 was potently prevented in the presence of 18-α-GA in culture medium (Figure 4F). These data suggest that the bystander effect of the combined treatment of lenti-*LEP503*-EGFP-HSV-tk/GCV system and ATRA on HLECs is significantly enhanced compared to treatment with lenti-*LEP503*-EGFP-HSV-tk/GCV alone, and that the bystander effect is primarily mediated through GJIC.

### The bystander effect results predominantly in cell necrosis

**Figure 5:**
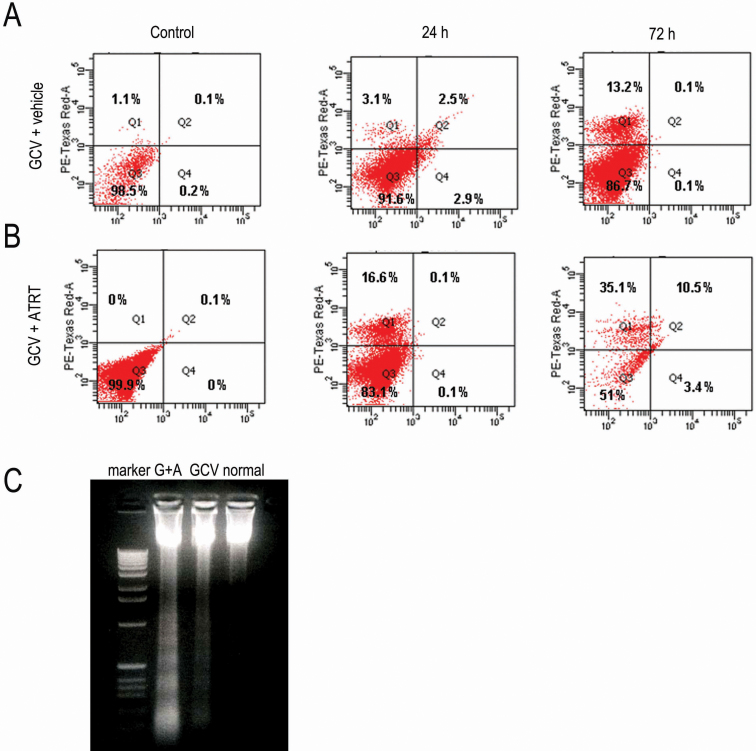
Cell necrosis is predominant phenotype in HLECs due to the bystander effects of combined treatment with the lenti-*LEP503*-HSV-tk/GCV system and ATRA. **A**: The analysis of necrotic or apoptotic phenotype of HLECs exposed to lenti-*LEP503*-HSV-tk/GCV system combined with vehicle control with flow cytometry assay. **B**: The analysis of necrotic or apoptotic phenotype of HLECs exposed to lenti-*LEP503*-HSV-tk/GCV system combined with ATRA with flow cytometry assay. **C**: Apoptosis in HLECs exposed to lenti-*LEP503*-HSV-tk/GCV system combined with ATRA with DNA ladder assay. G + A, Lenti-*LEP503*-HSV-tk/GCV + ATRT treatment; GCV, Lenti-*LEP503*-HSV-tk/GCV + vehicle treatment.

We investigated whether the cell killing was due to apoptosis or necrosis by determining the cell death type with a flow cytometry assay. The percentage of HLECs distributed in the Q1 zone (necrosis) was ~16.6% at 24 h and ~35.1% at 72 h after the combined treatment with the lenti-*LEP503*-HSV-tk-EGFP/GCV system with ATRA (Figure 5B), whereas the percentage in Q1 zone was ~3.1% at 24 h and ~13.2% at 72 h when cells were exposed to the lenti-*LEP503*-HSV-tk-EGFP/GCV system alone (Figure 5A). However, more cells (~10.5%) were located in Q2 zone (apoptosis) at 72 h after combined treatment with the lenti-*LEP503*-HSV-tk-EGFP/GCV system with ATRA (Figure 5B) than after treatment with the lenti-*LEP503*-HSV-tk-EGFP/GCV system alone (0.1%; Figure 5A). The HLECs treated with the combination of the lenti-*LEP503*-HSV-tk-EGFP/GCV system with ATRA also showed more typical DNA ladders than did cells exposed to the lenti-*LEP503*-HSV-tk-EGFP/GCV system treatment alone (Figure 5C). These data suggest that the bystander effect induced by combination of the lenti-*LEP503*-HSV-tk-EGFP/GCV system with ATRA inhibited HLEC proliferation predominantly by inducing cell necrosis. However, apoptosis also contributed to the bystander effect, although apoptotic cells comprised a minor proportion of the cells, consistent with our previous report [11].

## Discussion

PCO is the most common complication after cataract surgery. A clinical survey suggested that the incidence of PCO is not declining, in spite of improvements in surgical techniques and in the designs of intraocular lenses [24]. PCO currently is treated by Nd:YAG capsulotomy, which carries a small risk of sight-threatening complications such as cystoids macular edema, retinal detachment, and increased intraocular pressure [25]. Many attempts have been made to prevent PCO during or after surgery by removing as much of the LECs as possible, through the introduction of heparin and anti-inflammation medicines such as steroids into the irrigation fluid and by using an antimetabolite such as mitomycin or 5-fluorouracil. However, none of these methods is completely effective at preventing PCO. One candidate strategy for preventing PCO would be promotion of proliferation and death of lens epithelial cells without causing other cytotoxic effects in the body. In this case, suicide gene therapy makes sense. In a previous study, we reported the feasibility of killing HLECs by lentivirus-mediated HSV-tk/GCV as a way to prevent PCO [10,11]. Because of the non-selectivity of *CMV* promoter, we cloned the *LEP503* promoter, which specifically initiates gene expression in HLECs, and sub-cloned it into the HSV-tk/GCV vector. In the present study, we found that the *LEP503* promoter could drive the HSV-tk/GCV system in HLECs with relative specificity (Figure 2), consistent with our previous reports [10,11].

We also endeavored to improve the therapeutic efficacy of the targeted suicide gene system through the use of different clone strategies, special promoters, and in combination with other reagents. In the present study, we generated the lenti-*LEP503*-HSV-tk/GCV vector and examined its in vitro efficacy in HLECs when combined with ATRA. We observed that the *LEP503* promoter provides a relatively specific expression in HLECs (Figure 2), but was unable to provide high expression levels of the target gene (Figure 2). In addition, we found that the lenti-*LEP503*-HSV-tk/GCV system specifically induces a cell killing effect in HLECs and that the maximum inhibition was ~32% at 72 h after GCV treatment (Figure 3). The expression of HSV-tk induced by the lens-specific promoter *LEP503* was lower than that by the *CMV* promoter, so we reasoned that an effective alternative strategy might be to combine the vector with another reagent that could inhibit lens epithelial cell proliferation. Addition of ATRA significantly enhanced the sensitivity of infective and non-infective cells to the HSV-tk/GCV system [17]. Therefore, we speculated that the lentivirus-based *LEP503*-HSV-tk/GCV system induced a tropism for specific killing HLECs after delivery. Accordingly, we observed that the therapeutic bystander effect of lenti-*LEP503*-HSV-tk/GCV in HLECs was enhanced by combination with ATRT administration (Figure 4A,B,F), and by upregulating the GJIC through simultaneous overexpression of Cx43 in HLECs (Figure 4C,D). Our comparative experiments demonstrated that the inhibitory effect was ~32% only 24 h after GCV and ATRA treatment, whereas similar inhibitory effects required 72 h with GCV treatment alone (Figure. 4A) The maximum inhibition was ~71% at 72 h after GCV and ATRA treatment (Figure 4A), which was significantly higher than the inhibition (~32%) seen with GCV alone. In addition, we used GCV at a concentration of 20 μM, rather than a higher dose, and were able to avoid cytotoxic effects of the reagent. The novel strategy of combination with other reagents could provide benefits to HSV-tk/GCV gene therapy. Several studies have investigated the association of suicide genes with other therapeutic reagents, and similar positive results were obtained both in vitro and in vivo [3,5,26].

The key to success of gene delivery systems is the bystander effect, which relies on the ability of transfected cells to kill neighboring untransfected targeted cells. Several mechanisms may be responsible for this bystander effect, including transfer of apoptotic vesicles, exocytosis of cytotoxic factors, or an enhanced cellular immune response [27]. However, the most common view is that this effect is exerted mainly through interactions with GJIC. Our result showing the increased alteration of Cx43 of GJIC by ATRA treatment suggests a direct enhancement of the bystander effect, which would be beneficial for suicide gene therapy against PCO (Figure 4C,D) [25,26,28]. This finding also supports the hypothesis that gap junctions allow the passive diffusion of the activated metabolites into neighboring cells, and inducing GCV cytotoxicity in the genetically altered cells as well as non-transfected neighboring cells. This observation was further supported by co-culture assays in which 18-α-GA, an inhibitor of GJIC, prevented the bystander killing effect under co-culturing conditions (Figure 4F), consistent with other reports [29].

The GJIC plays an important role in facilitating metabolic cooperation in normal cells and in many malignant cell types, although they are present at low levels or even completely absent from tumor cells [30]. The bystander effect relies primarily on the transfer of phosphorylated GCV molecules between cells via gap junctions [31]; i.e., phosphorylated GCV passes from HSV-tk gene-positive cells to negative neighboring cells via gap junctions [32].

ARTA, a physiologic metabolite of vitamin A, plays an important role in a broad spectrum of biologic processes, including inhibition of proliferation, induction of differentiation, regulation of apoptosis, and control development [33,34]. Due to its strong anti-proliferative activity and relatively low toxicity, ATRA represents an attractive option for chemoprevention and treatment of human malignancies. It is currently used to ameliorate various models of autoimmunity [35-37] and to treat promyelocytic leukemia [38] and some epithelial cancers [39]. ATRA is an ideal chemical inducer of GJIC and has a wide range of biologic actions, including an ability to cross the blood–brain barrier without causing any harm to the body. ATRA is an effective drug against acute promyelocytic leukemia, with weak side effects [38]. Through binding to its receptors and a post-translational mechanism of action, ATRA antagonizes the effects of two serine/threonine protein kinase families: protein kinase C and MAPK. This results in a in phosphorylation of Cx43 and (or) other connexin proteins, such as Cx26 [40,41], and upregulates expression of Cx43 in a variety of cells [42]. In our studies, ATRA significantly increased the expression of Cx43 in HLECs (Figure 4C,D), and significantly enhanced the bystander killing effect of the lenti-LEP503-HSV-tk/GCV system in HLECs (Figure 3 and Figure 4), although we have not yet evaluated the expression of Cx26.

ATRT is also an effective dermatological treatment agent. However, its topical use is limited by its poor skin absorption and it also causes undesirable adverse effects known as retinoid dermatitis on the treated area, which includes irritation, erythema, stinging, itching, burning, and desquamation [20]. An effective delivery system or therapeutic formulation may therefore be helpful [20]. One well known side effect is ATRT syndrome [38], when ATRA is used as a clinical treatment for acute promyelocytic leukemia. Occasionally, other life threatening conditions was reported, such as reactive hemophagocytosis [43]. However, from the perspective of physiology and anatomy, the eyeballs are relatively independent organs [44]. Therefore, we assume that topical administration to the eyeballs may have far less side effects than application to other areas.

Our findings suggest that a HSV-tk/GCV suicide gene therapy system, controlled by the relatively specific *LEP503* promoter and combined with ATRA as an adjuvant, may have important implications for PCO treatment by in vivo killing of residual HLECs. Potential side effects of ATRA should be reduced by the use of the HSV-TK/GCV suicide gene system because a much smaller effective dose will be needed and topical administration to the eyes can be used for PCO. Further studies on in vivo effects of the PCO treatment are needed if this is to be used as a preventive treatment; i.e., the lenti-*LEP503*-HSV-tk-EGFP/GCV system with ATRA should be locally administered as soon as possible after cataract surgery. As previously described, viral vector suspensions and other reagents were injected with a 30 G needle into the anterior chamber of eyes of experimental rabbits [16]. More work is needed for the design of effective therapeutic interventions.

In conclusion, the present study supports the idea that the bystander effect ATRA, combined with delivery of the Lenti-*LEP503*-HSV-tk/GCV system, could be an effective PCO treatment. The mechanism appears to be killing of the proliferative HLECs by enhancing the function of GJIC, although this needs further confirmation in vivo experiments. The use of a selective promoter such as the *LEP503* will also help to restrict therapy to specific cells, avoiding other cell types. The bystander effect could provide additional beneficial effects to that of promoter selectivity by killing neighboring, but uninfected, target cells [29]. Clinical use of this type of biologic therapeutic regimen will require more research on the toxicity of the system. Formulation of the correct combination of therapeutic regimens, prediction of curative effects, and appraisal of prognosis in animal experiments are also essential.
